# A family with *PTEN* mutations with malignancy and an unusually high number of offspring with autism spectrum disorder: a case report

**DOI:** 10.1186/s13256-018-1863-0

**Published:** 2018-11-28

**Authors:** Sabrina L. Gruhl, Pankaj Sharma, Thang S. Han

**Affiliations:** 1 St George’s Hospital Medical School, University of London, London, SW17 0RE UK; 2 Institute of Cardiovascular Research, Royal Holloway, University of London, Egham, Surrey TW20 0EX UK; 3 Department of Clinical Neuroscience, Imperial College London NHS Trust, London, SW7 2AZ UK; 4 Department of Endocrinology, Ashford and St Peter’s NHS Foundation Trust, Surrey, KT16 0PZ UK

**Keywords:** Cowden’s syndrome, PTEN hamartoma tumor syndrome, Mucocutaneous manifestations

## Abstract

**Background:**

Cowden’s syndrome (OMIM:158350), a rare genetic disorder (incidence ~ 1:250,000), is caused by mutations of the tumor suppressor gene *PTEN*. In this report, we describe clinical manifestations of a 56-year-old patient diagnosed with Cowden’s syndrome and his family with *PTEN* mutations. The family has an unusually high number of offspring with autism spectrum disorder.

**Case presentation:**

Except for his 80-year-old Caucasian father, all of our index case’s living Caucasian kindred (three children, brother, and nephew) had *PTEN* mutations and macrocephaly. Prior to genetic testing, his mother and sister died of breast cancer at 42 and 38 years old, respectively. After *PTEN* mutation was identified, our patient underwent complete thyroidectomy (histology showing micropapillary carcinoma) and right nephrectomy for renal cell carcinoma. All of his three children (13-year-old son, 11- and 8-year-old daughters) have been diagnosed with autism spectrum disorder. His son and brother underwent total thyroidectomy. His nephew had thyroid nodules. Management of Cowden’s syndrome requires clinical examinations and investigations every 6 to 12 months from 18 years old or 5 years before the family’s earliest age of cancer diagnosis and should focus on all clinical manifestations associated with *PTEN* mutations to identify early abnormal changes in skin, breasts, thyroid, endometrium, gut, and kidneys. Input from specialists across different disciplines is necessary.

**Conclusions:**

We describe a man and his family with *PTEN* mutations who have increased risk of cancers and an unusually high number of offspring with autism spectrum disorder. Early recognition and close surveillance are vital in order to provide treatment and early screening for asymptomatic at-risk relatives.

## Background

Cowden’s syndrome (OMIM:158350), a rare autosomal dominant disorder with an incidence of about 1 in 250,000 [1], is a clinically distinct syndrome of PTEN hamartoma tumor syndrome (PHTS) [2]. Cowden’s syndrome was first described by Lloyd and Dennis in 1963 [3], who detailed the phenotypic findings in a 20-year-old patient, Rachel Cowden, after whom the condition was named (originally Cowden’s disease). Cowden’s syndrome is caused by germline mutations in the tumor suppressor gene *PTEN* (phosphate and tensin homologue deleted on chromosome 10) mapped to 10q22–23 [4]. Over 300 mutations have been identified so far [5, 6]. These mutations include changes in a small number of base pairs, but deletions of a larger amount of genetic material from the *PTEN* gene may at times occur, leading to the production of a dysfunctional PTEN enzyme. The defective enzyme is unable to inhibit apoptosis or signal abnormal cells to die, which contributes to cell proliferation.

Cowden’s syndrome is characterized by multiple hamartomas with an increased risk of benign or malignant tumors. Mucocutaneous manifestations (primarily trichilemmomas and papillomatous papules) along with acral keratoses are pathognomonic features that exhibit age-related penetrance and are present in almost all individuals with Cowden’s syndrome by their third decade of life. Other features commonly observed in patients with Cowden’s syndrome include Lhermitte-Duclos disease, megencephaly, macrocephaly, dolicocephaly, gastrointestinal polyps, glycogenic acanthosis [7], and tumors, with breast cancer being the most common form of malignancy, followed by thyroid and endometrial cancer.

Diagnosis of Cowden’s syndrome is based on National Comprehensive Cancer Network criteria [8]. The diagnostic criteria are subdivided into pathognomonic, major, and minor categories. The clinical diagnosis can be made on the basis of pathognomonic mucocutaneous lesions alone if an individual exhibits one of the following: (1) six or more facial papules (three of which must be trichilemmomas), (2) cutaneous facial papules and oral mucosal papillomatosis, (3) oral mucosal papillomatosis and acral keratosis, or (4) six or more palmoplantar keratoses. Macrocephaly along with breast, thyroid, and endometrial cancer make up the four major criteria. Minor criteria are composed of other thyroid lesions (adenoma and multinodular goiter), mental retardation, hamartomatous intestinal polyps, fibrocystic breast disease, lipomas, fibromas, genitourinary tumors (particularly renal cell cancer), genitourinary malformations, and uterine fibroids, which are more weakly associated with Cowden’s syndrome. If an individual has a first-degree relative with Cowden’s syndrome, the criteria require only one of the following: any single pathognomonic or major criterion, two minor criteria, or history of Bannayan-Riley-Ruvalcaba syndrome (BRRS). After Cowden’s syndrome is established clinically, the actual diagnosis of PHTS is confirmed only by the identification of a germline mutation in *PTEN*/*MMAC1*/*TEP1* [8].

Parents with *PTEN* mutations have been observed to have a disproportionately high number of offspring with autism spectrum disorder (ASD) and macrocephaly [9, 10], but only a handful of reports have described families with Cowden’s syndrome and ASD [11, 12], and those reports that have been documented with pedigrees tend to be small and incomplete.

In this report, we describe a case of Cowden’s syndrome with a strong family history of multiple malignancies and an unusually high number of members with ASD in a very clearly phenotyped family.

## Case presentation

Our patient was a 56-year-old Caucasian father (index case) of three children was previously diagnosed with Cowden’s syndrome (*see below*). The patient was referred to our endocrinology department by his oncology geneticist for management of his thyroid cancer in 2014.

**Fig. 1:**
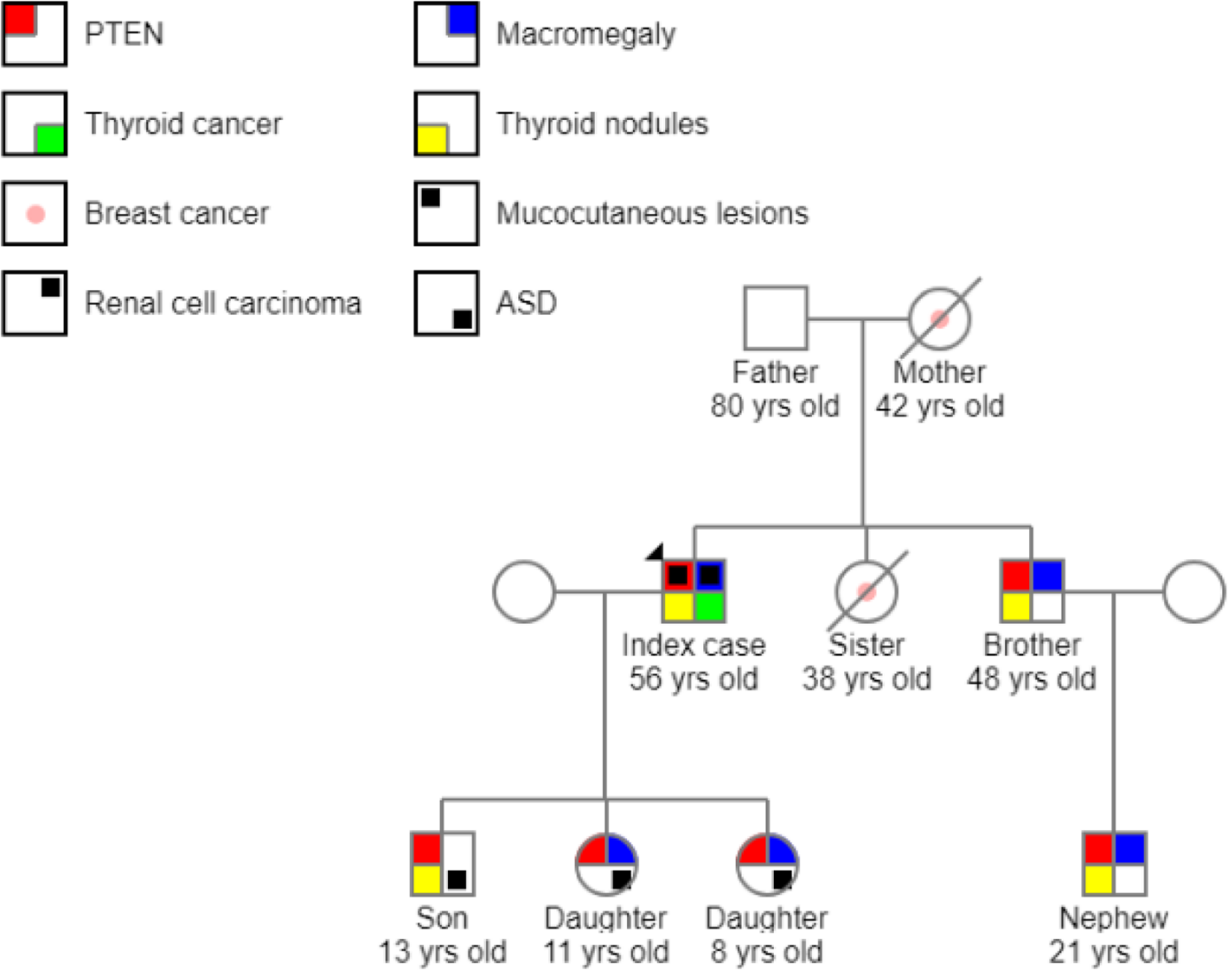
Pedigree chart showing clinical manifestations in a family with *PTEN* mutations inherited in an autosomal dominant pattern. Mother and sister of index case died before genetic testing was performed for the family; therefore, their *PTEN* status is unknown

In 2009, when his 5-year-old son was undergoing investigations for ASD, the father was asked to have a blood test for genetic screening because the child was too anxious. The father was found to carry a C→G substitution at base 176, c.176C>G in exon 3 of the *PTEN* gene, changing amino acid number 59 from serine to a stop codon, p.S59X. Thereafter, genetic testing was carried out for all immediate members of his family. Except for the patient’s 80-year-old father, all of his living kindred (three children, brother, and nephew) were found to carry *PTEN* mutations. His mother and sister had died of breast cancer at 42 and 38 years old, respectively, prior to family genetic testing (Fig. 1).

Before his diagnosis of Cowden’s syndrome, the patient had undergone nasal polypectomy in 2004 and two subtotal thyroidectomies in 1987 and 2002 (histology showed papillary thyroid carcinoma). In view of his increased risk of thyroid cancer due to *PTEN* mutations, the patient was referred to a head and neck surgeon and advised to undergo complete thyroidectomy in 2014 (histology revealed micropapillary carcinoma).

At the time of review, he was taking two tablets of Calcichew D_3_ (Takeda UK, Wooburn Green, UK) and 150 μg of levothyroxine daily, which adequately suppressed his thyroid-stimulating hormone (TSH) levels and kept his thyroglobulin at an undetectable level, indicating no disease recurrence. Hematological and biochemical assessments showed a hemoglobin of 14.4 g/dl, creatinine 73 μmol/L, calcium 2.15 mmol/L, alanine aminotransferase 27 IU/L, TSH 0.26 mU/L, free thyroxine 19.8 pmol/L, thyroglobulin < 0.2 μg/L, and vitamin D 43 nmol/L. The patient never smoked or consumed alcohol. He is currently living with his wife and three children and works as a bus driver. His physical examination showed evidence of macrocephaly, a neck scar from a previous thyroidectomy, palmoplantar keratoses, and mucosal papillomas on the tongue. He had no focal neurological deficits. His weight was 101 kg, and his height was 1.74 m (body mass index 33 kg/m^2^). His blood pressure was 130/80 mmHg, and his heart rate was 68 beats per minute in regular rhythm.

**Fig. 2:**
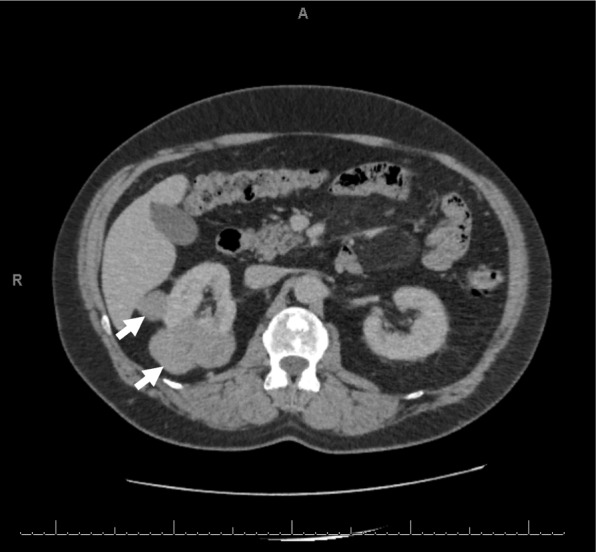
Computed tomographic scan of kidneys showing tumor in the right kidney (*white arrows*) prior to nephrectomy

Ultrasound surveillance in 2018 revealed two masses in his right kidney that were confirmed by computed tomography (CT) (Fig. 2). CT-guided biopsy showed chromophobe-type renal cell carcinoma, and laparoscopic right nephrectomy was performed.

The patient has been regularly monitored for manifestations associated with *PTEN* mutations according to National Comprehensive Cancer Network guidelines [8]. His palmoplantar keratoses and the mucosal papillomas on his tongue are being monitored by a dermatologist. He is currently attending a polyposis clinic and is under the care of an upper gastrointestinal team for gastrointestinal surveillance. Colonoscopy is being performed biannually, and in a recent colonoscopy, two benign 3-mm cecal polyps were excised.

A number of clinical manifestations arising from *PTEN* mutations have emerged among his kindred. His now 13-year-old son and 11- and 8-year-old daughters have been diagnosed with ASD. The patient’s son, brother, and nephew developed thyroid nodules; both the son and brother underwent total thyroidectomy (nonmalignant). The son also has skin lesions on his back (currently being monitored by a dermatologist). The patient and all members of his family with *PTEN* mutations have macrocephaly (Fig. 1). His 90-year-old maternal aunt did not undergo genetic testing. His other maternal aunt died at the age of 70 prior to our patient’s diagnosis with Cowden’s syndrome. None of the patient’s maternal aunts had a history of cancer.

The patient, his mother, and his brother all completed secondary school and work as bus drivers. Before dying, his mother had been a full-time house wife and his sister had graduated from a university and worked as an accountant. His nephew also completed a university degree. All his children are attending a school for children with special needs (Table 1).

**Table 1:** Educational attainment and occupation of patient and his family with *PTEN* mutation or with history of cancer

	Age (years)	*PTEN* mutation status	Highest education attainment	Occupation
Patient (index case)	56	Positive	Secondary school	Bus driver
Mother	Died at 42	Unknown (died of breast cancer)	Secondary school	Housewife
Sister	Died at 38	Unknown (died of breast cancer)	University	Accountant
Brother	48	Positive	Secondary school	Bus driver
Son	13	Positive	School for children with special needs	–
Daughter	11	Positive	School for children with special needs	–
Daughter	8	Positive	School for children with special needs	–
Nephew	21	Positive	Currently studying at university	–

## Discussion

It has been recognized that parents with *PTEN* mutations have a disproportionately high number of offspring with ASD [9, 10], but there are only a few reports on families with Cowden’s syndrome and ASD [11, 12]. In this report, we describe a case of a patient with Cowden’s syndrome who has a strong family history of cancers and a variety of manifestations from *PTEN* mutations. Although germline *PTEN* pathogenic variants have been reported to be present in 10–20% of individuals with ASD [9, 12–14], little information is available on the prevalence of ASD among families with Cowden’s syndrome. The high proportion of offspring with ASD in our patient’s case (all three children) is unusually high for a single family. The patient himself has exhibited progressive development of tumors with age, suggesting the importance of close health monitoring for this group of patients.

Before gene identification was available, patients with Cowden’s syndrome were rarely diagnosed. The only available estimate of population frequency is 1:250,000 [1], which is likely to be conservative because of its variable expression and subtle mucocutaneous lesions, as exemplified by our patient, who was found to carry a *PTEN* mutation only through investigation of his son for ASD. He therefore underwent several subtotal thyroidectomies prior to his diagnosis with Cowden’s syndrome. A delay in diagnosis clearly has a devastating impact on at-risk members of the family, as demonstrated in this report: His mother and sister both developed breast cancer and died of this condition. Members in the younger generation of his family are now closely monitored for early features of PHTS, including thyroid ultrasound.

Thyroid abnormality occurs in all male members with *PTEN* mutations in this family (our patient and his son, brother, and nephew), which is one of the most frequently reported manifestations of Cowden’s syndrome, affecting between two-thirds and three-fourths of patients. These include benign thyroid abnormalities, such as multinodular goiter, adenomatous nodules, and follicular adenomas, whereas there is a 3–10% increase in lifetime risk of thyroid cancer, such as follicular and papillary thyroid cancer, but never medullary cancer [15].

Both first-degree adult female relatives (mother and sister) of our patient developed breast cancer, a common manifestation of Cowden’s syndrome in women, and they died of this disease at young ages (38 and 42 years). Although they did not have the opportunity to undergo genetic testing, given the negative test for *PTEN* mutations in his father, the young age at the time of their death, and the autosomal dominant inheritance of Cowden’s syndrome, it is likely that his mother and sister also carried *PTEN* mutations. Women with Cowden’s syndrome have a lifetime risk of developing benign breast disease of about 67% and of breast cancer between 25% and 50%, with an average age at diagnosis between 38 and 46 years [16, 17]. Men with Cowden’s syndrome have also been shown to have breast cancer [18]. About half of women with Cowden’s syndrome have been shown to develop multiple large uterine fibroids and have a lifetime risk of developing endometrial cancer between 5% and 10%.

Our patient also has other manifestations of Cowden’s syndrome, including renal cell carcinoma, gastrointestinal and nasal polyps, palmoplantar keratoses, and mucosal papillomas, which are cardinal features of Cowden’s syndrome. The risk of other cancers is also increased among individuals with Cowden’s syndrome, including skin and possibly brain tumors.

Macrocephaly was found in all family members with *PTEN* mutations in the present report. Authors of a previous report described a 14-year-old boy with *PTEN* mutations who had progressive macromegaly, ASD, and focal epilepsy [19]. Studies using magnetic resonance imaging of the brain have shown that macrocephaly in patients with ASD who carry a *PTEN* mutation is due to overgrowth of the white matter [20, 21], but how changes in the *PTEN* gene are related to the risk of developing ASD remains to be elucidated.

In their study of *PTEN* mutation spectrum and genotype-phenotype correlations in BRRS and Cowden’s syndrome, Marsh *et al.* showed the overlap of a number of clinical features, the sharing of identical *PTEN* mutations, in addition to the presence of BRRS/Cowden’s syndrome overlap families, all highly suggestive that BRRS and Cowden’s syndrome are different presentations of a single syndrome and that anticipation may also pertain in this syndrome [22]. However, there has been no evidence from subsequent studies to support this notion. It is of interest that our patient’s family appears to have increasing severity of learning difficulty with the youngest generation, as reflected by education level and high frequency of ASD among offspring.

### Management of Cowden’s syndrome

Annual clinical examinations are recommended between 6 and 12 months from 18 years old or 5 years before the family’s earliest age of cancer diagnosis. Examinations should focus on all clinical manifestations associated with *PTEN* mutations, including skin, breast, thyroid, endometrial, gastrointestinal, and renal cell cancers. This would require input from specialists across different disciplines. Up-to-date management guidelines are available from the National Comprehensive Cancer Network [8].

The strengths of this case report lie in its details of the clinical manifestations of all immediate family members and the relatively large number of offspring in a single family, all of whom have ASD. This case is limited by the reliance on clinical history elicited from one member of the family (the index case). We overcame this limitation by conducting thorough research of the medical case notes and communicating with specialists in charge of the patient and his family.

## Conclusions

We describe a man and his family with *PTEN* mutations who have increased risk of cancers and an unusually high number of offspring with ASD. Early recognition and close surveillance are vital in order to provide treatment and early screening for asymptomatic at-risk relatives.

## Abbreviations

ASD: Autism spectrum disorder
BRRS: Bannayan-Riley-Ruvalcaba syndrome
CT: Computed tomography
PHTS: PTEN hamartoma tumor syndrome
*PTEN*: Phosphate and tensin homologue deleted on chromosome 10
TSH: Thyroid-stimulating hormone
